# Prophylactic Phenylephrine Increases the Dose Requirement of Oxytocin to Treat Uterine Atony During Cesarean Delivery: A Double-Blinded, Single-Center, Randomized and Placebo-Controlled Trial

**DOI:** 10.3389/fphar.2021.720906

**Published:** 2021-10-20

**Authors:** Yao-Hua Shen, Fan Yang, Li-Dan Jin, Yu-Jia Qian, Li Xing, Ya-Li Huang, Su-Feng Lin, Fei Xiao

**Affiliations:** ^1^ Department of Anesthesia, Hangzhou City Linping District Maternal and Child Care Hospital, Hangzhou, China; ^2^ Key Laboratory for the Genetics of Developmental and Neuropsychiatric Disorders (Ministry of Education), Bio-X Institutes, Shanghai Jiao Tong University, Shanghai, China; ^3^ Department of Anesthesia, Jiaxing University Affiliated Women and Children Hospital, Jiaxing, China

**Keywords:** phenylephrine, oxytocin, cesarean delivery, uterine tone, hypotension

## Abstract

**Purpose:** Studies involving mouse models and human uterine smooth muscle cells have shown that phenylephrine inhibits uterine contractions in non-pregnant mice and human *in vitro* cell *via* cyclic adenosine monophosphate (cAMP) signaling. However, there has been no limited exploration to date of the effect of phenylephrine on uterine contractions in clinical practice. This study aimed to compare the dose requirement of oxytocin with or without the infusion of prophylactic phenylephrine to prevent post spinal hypotension during cesarean delivery under combined spinal and epidural anesthesia.

**Methods:** This was a double-blinded, single-center, randomized, control study. One hundred and sixty pregnant patients provided informed consent and were randomly allocated to the phenylephrine (phenylephrine infusion) and control (saline infusion) groups. Patients randomized to the phenylephrine group received an intravenous prophylactic phenylephrine infusion at a fixed rate of 0.5 μg/kg/min. The control group received a saline placebo at the same rate and used the same apparatus for delivery. After neonatal delivery and clamping of the umbilical cord, patients received a standard institutional oxytocin protocol. The primary outcome measure was the total dose of oxytocin administered during CD. Secondary outcomes including the proportion (%) of patients requiring a secondary uterotonic agent and estimated blood loss (EBL) in the first 24 h after surgery.

**Results:** The median oxytocin dose administered was significantly higher in the phenylephrine group than in the control group [6.9 ± 2.5 international standardized units (IU) *vs.* 5.4 ± 2.4 IU, *p* = 0.0004]. The number of patients that required a secondary uterotonic agent was significantly higher in the phenylephrine group than in the control group (24.2% *vs.* 9.1%; *p* = 0.034). The EBL in the first 24-h postoperatively was similar between the two groups (467 ± 47 ml *vs.* 392 ± 38 ml; *p* = 0.22).

**Conclusions:** Prophylactic infusion of phenylephrine used to prevent post-spinal hypotension during CD was associated with a higher dose of oxytocin. This has important clinical implications, as the suboptimal use of oxytocin is associated with an increased risk of postpartum hemorrhage and increased maternal morbidity and mortality. Further studies are now needed to confirm these findings.

## Introduction

Hypotension is the most common side effect of spinal anesthesia during cesarean delivery (CD) and is associated with increased maternal and neonatal morbidity (NganKee, 2010; Gizzo et al., 2014). Treating or preventing post-spinal hypotension is therefore a care priority for obstetric patients. Phenylephrine is widely used by anesthesiologists worldwide for both the treatment or prevention of post-spinal hypotension and is associated with a lower incidence of fetal acidemia than ephedrine (NganKee et al., 2009; Heesen et al., 2014; NganKee, 2017; Kinsella et al., 2018).

However, there are several adverse events associated with the routine use of phenylephrine during obstetric anesthesia; both bradycardia and a corresponding reduction cardiac output (CO) have been widely reported (NganKee, 2017; Kinsella et al., 2018). Norepinephrine, which has weak *β*-adrenergic receptor agonist activity, has been suggested as an alternative vasopressor, but has yet to be universally adopted (NganKee et al., 2015; Puthenveettil et al., 2019; Sharkey et al., 2019). Recent murine studies have demonstrated that phenylephrine inhibits uterine contractions in non-pregnant mice through cAMP signaling (Chen et al., 2018). In addition, in Chen et al. (2018) study, they also observed an increase in cAMP induced by phenylephrine in human uterine smooth muscle cells (HUSMCs), thus suggesting that phenylephrine may also exhibit antagonistic effects against uterine contractions in the human uterus. Although this was an *in vitro* trial and needs to be confirmed by *in vivo* studies, this effect is still worth the vigilance of obstetric anesthesiologists. Furthermore, there is a lack of evidence for this effect in clinical practice, the routine use of phenylephrine in obstetric anesthesia to prevent post spinal hypotension may increase the risk of uterine atony, and subsequent rates of postpartum hemorrhage.

In this randomised and controlled trial, we aimed to compare the dose requirement of intravenous oxytocin between patients receiving an infusion of prophylactic intravenous phenylephrine to those receiving a saline placebo control infusion under combined spinal and epidural anesthesia (CSEA). The primary outcome of this study was the mean dose of oxytocin in the perioperative period. The secondary outcome of this study was the estimated volume of blood loss, the use of secondary uterotonic agents and the side effects during CD. Our hypothesis was that prophylactic phenylephrine may increase the dose requirement of oxytocin to prevent uterine atony.

## Materials and Methods

### Ethics

Ethical approval for this study (Ethical Committee No. LLSC-KYKT-2020-0013-A) was provided by the Ethical Committee of Hangzhou City Linping District Maternal and Child Care Hospital, Hangzhou, China (Chairperson Prof Yue-jian Shen) on February 1, 2020. The study was also registered on the Chinese Clinical Trial Register (ChiCTR2000030884) on March 16, 2020. The trial was conducted in a single center, Hangzhou City Linping District Maternal and Child Care Hospital. All patients involved in this study provided written, signed, and informed consent before inclusion. The first patient was enrolled in this study on the March 20, 2020.

### Design

This was a double-blinded, single-center, randomized, control study.

### Patients and Setting

The patient inclusion criteria were as follows: single pregnancy, at full-term (gestational age ≥37 weeks), American Society of Anesthesiologists Physical Status II, aged between 18 and 40 years old, undergoing elective CD and planning epidural anesthesia. The exclusion criteria included obesity (body mass index, BMI >35 kg/m^2^), height (<150 or >170 cm), active labor, early labor, diabetes mellitus or gestational diabetes, hypertension or pre-eclampsia, intrauterine growth restriction or fetal macrosomia, placenta previa, placenta conglutination, placenta implantation, significant co-existing maternal disease, any absolute contraindications to spinal or epidural anesthesia such as local infection or bleeding disorders, and cases with a sensory plane block that did not reach T6 or higher.

### Study Protocol

Patients were randomly allocated to the phenylephrine intervention or placebo control group by using a randomization schedule generated by YH Shen using Microsoft Excel (Microsoft Corporation, Redmond, WA, United States); this investigator knew the patient groupings but did not participate in patient care or data collection. The randomization schedule was kept in numbered opaque envelopes, that were opened sequentially with each patient enrollment. All study participants were blinded to the study design and their randomised allocation. To maintain investigator blinding, phenylephrine and saline were prepared in identical 50-ml infusion syringes (lettered A or B) and the infusion rate was set at 50 ml/h (equal to 0.5 μg/kg/min in on our prior data) (Xiao et al., 2020).

Upon arriving at the operating room, peripheral access was obtained using an 18 G intravenous cannula inserted into an upper limb vein. Oxygen was delivered *via* a nasal catheter at the rate of 3 L per minute. Standard monitoring was applied including a non-invasive blood pressure cuff, pulse oximeter, and electrocardiography leads (including a respiratory rate monitor). The patient’s baseline systolic blood pressure (SBP) and heart rate (HR) were established by calculating the mean of 3 consecutive measurements at 3-min intervals with the patient at rest. Intrathecal 15 mg hyperbaric ropivacaine with 5 μg sufentanil was injected into the subarachnoid space (over 20 s) after the emergence of cerebrospinal fluid (CSF). Spinal anesthesia was administered using a needle-through-needle technique of combined spinal epidural anesthesia (CSEA) at the L3-L4 disc space by palpation with the patient in a left lateral position. An infusion of 20 ml/kg/h of warmed Lactated Ringer’s solution was also administered intravenously at this time.

Patients received either 50 ml/h of phenylephrine (0.5 μg/kg/min) in the intervention group or a saline placebo at the same rate, with the purpose of preventing post spinal hypotension at the time of intrathecal injection. Hypotension was defined as a decrease in baseline SBP ≥20%, or a fall of SBP <90 mm Hg. Where hypotension was detected, a co-infusion with 500 μg metaraminol was started. Hypertension, defined as a value >120% of baseline SBP, was treated with an infusion stop. Bradycardia was defined as a HR < 55 beats/min. If bradycardia was accompanied by hypotension, it was treated with an IV bolus of 0.5 mg atropine. If not, the infusion was paused and restarted when HR exceeded 55 beats/min.

Surgery began when the sensory plane of the block reached T6 or above. All caesarean procedures were performed by the same team of obstetricians. The adequacy of uterine tone (UT) was assessed by the same obstetrician (with 15 years of obstetric experience) at 3-min intervals up until the point when the obstetrician surgically closed the peritoneum (in accordance with existing literature) (Butwick et al., 2010; Lavoie et al., 2015; Duffield et al., 2017). After neonatal delivery and clamping of the umbilical cord, patients received a standard institutional oxytocin protocol, as follows. Specifically, patients received 3 IU of intravenous oxytocin as a loading dose (administered over 15 s). The patients were then assessed at 3-min intervals. If uterine tone was inadequate, then 3 IU of oxytocin was given intravenously as a rescue dose. If after three total doses of oxytocin (including the loading dose and 2 rescue doses), the UT was still regarded as inadequate, then secondary uterotonic agents were administered as needed (0.25 mg of intramuscular carboprost or 0.1 mg of intravenous carbetocin) (Kovacheva et al., 2015).

### Measurements

The primary outcome measure was the mean total dose of oxytocin used during CD. Secondary outcomes included the proportion (%) of patients requiring a secondary uterotonic agent, estimated blood loss in the 24 h after surgery (EBL), pH value of umbilical arterial blood, and adverse effects during surgery (hypotension, bradycardia, nausea and vomiting, flushing, chest pain, or dyspnea). EBL was estimated by the following formula (Carvalho et al., 2004; Sheehan et al., 2011) in accordance with previous literature: EBL (ml) = [(preoperative HCT–postoperative HCT)/preoperative HCT] × (weight in kilograms) × 85.

### Sample Size Estimation

Next, we used PASS software (version 11.0.7; NCSS, LLC, Kaysville, Utah, United States) to perform a power calculation. To detect a difference of 1.5 IU in total oxytocin administration between the intervention and control groups would require a minimum of 60 patients per group to achieve 80% power with an *α*-level of 0.05. Accounting for an attrition rate of 20%, the Ethical Committee permitted the recruitment of 80 patients per group.

### Statistical Analysis

Data are presented as mean ± SD for parametric data, median (and inter-quartile range) for non-parametric data, or *n* (%) as appropriate. The normality of continuous variables was assessed by the Kolmogorov-Smirnov test. Differences between normally distributed variables were tested using the Student’s *t* test, while differences for non-normally distributed variables were tested using the Mann-Whitney *U* test. Categorical variables were assessed using the Chi-squared test with Fisher’s exact modification where appropriate. Analyses were performed using IBM SPSS Statistics for Windows version 22.0 (IBM Corp, Armonk, NY) and GraphPad Prism version 5.0 (GraphPad Software Inc., San Diego, CA). *p* values <0.05 (two sides) were regarded as statistically significant.

## Results

**FIGURE 1:**
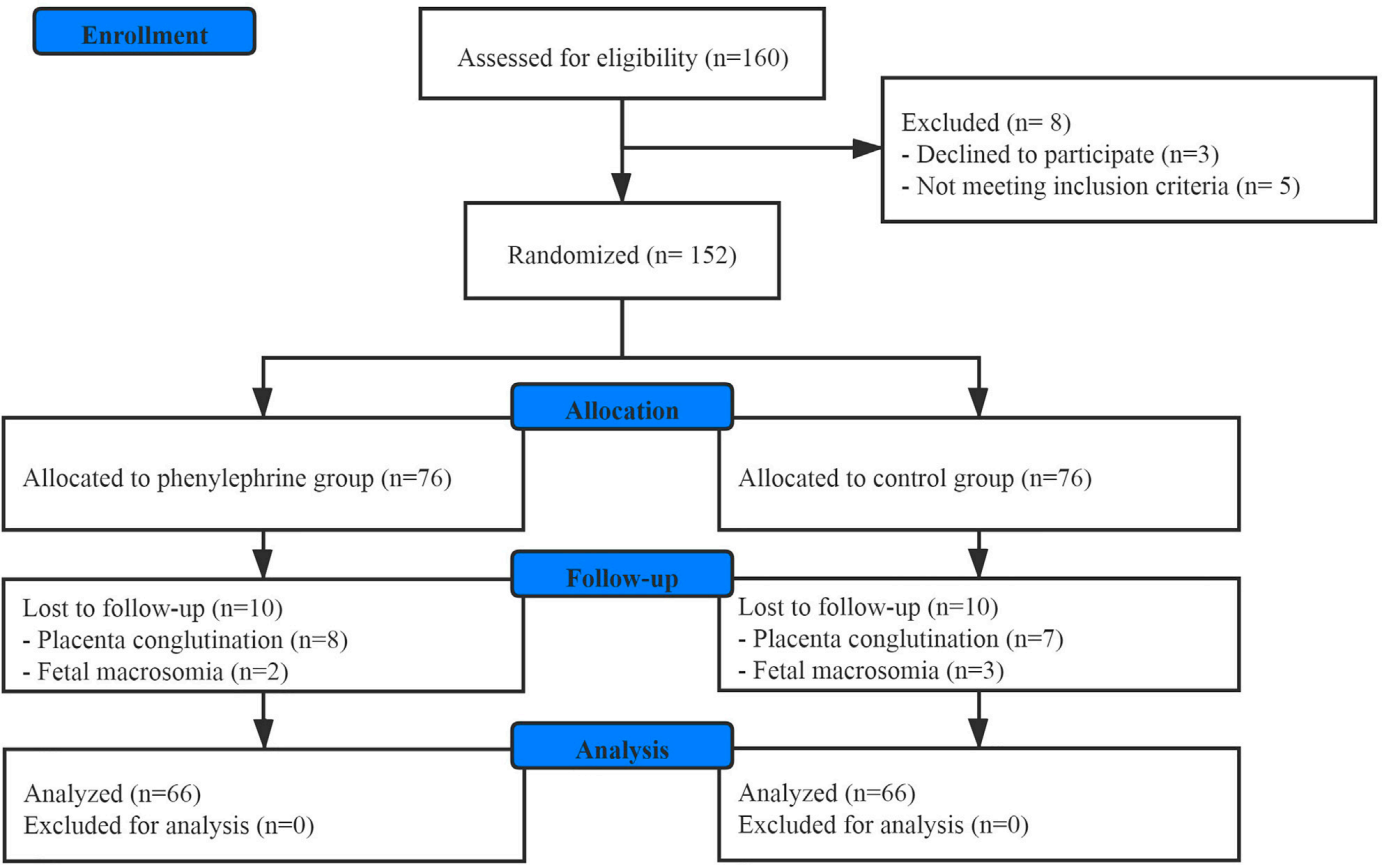
CONSORT diagram.

One hundred and sixty patients scheduled for elective CD were enrolled in this study. Finally, one hundred and thirty-two patients were involved in the final analysis (Figure 1). There were no significant differences between the groups with regards to baseline demographics, or the surgical duration (Table 1).

**TABLE 1:** Demographic data and surgical times.

	Phenylephrine group	Saline group	*p* Value
Age (yrs)	29.4 ± 3.8	29.1 ± 4.3	0.653
Height (cm)	159.2 ± 5.0	158.5 ± 5.0	0.445
Weight (kg)	69.6 ± 8.1	68.9 ± 7.8	0.643
BMI (kg/cm2)	27.4 ± 2.6	27.4 ± 2.6	0.980
Gestational age (weeks)	38.5 ± 0.8	38.4 ± 0.9	0.539
Fetal weight (g)	3,278 ± 302	3,326 ± 376	0.423
Cesarean history [*n* (%)]	44 (66.7)	45 (68.2)	0.853
Surgery time (min)	45.0 ± 13.3	45.8 ± 12.6	0.742

Data are presented as n (%), mean ± SD or median (IQR).

The mean oxytocin dose administered was significantly higher in the phenylephrine group than in the control group [6.9 ± 2.5 international standardized units (IU) *vs.* 5.4 ± 2.4 IU, *p* = 0.0004]. The number of patients that required a secondary uterotonic agent was significantly higher in the phenylephrine intervention group than in the saline placebo control group (24.2% *vs.* 9.1%; *p* = 0.034). The EBL in the first 24-h postoperative period was similar between the two groups (467 ± 47 ml *vs.* 392 ± 38 ml; *p* = 0.22) (Table 2).

**TABLE 2:** Comparison of oxytocin, secondary uterine agent, EBL, phenylephrine and rescued metaraminol dose and pH value of umbilical arterial blood.

	Phenylephrine group	Saline group	*p* Value
Oxytocin dose (IU)	6.9 ± 2.5	5.4 ± 2.4	0.0004
Secondary uterine agent [*n* (%)]	16 (24.2)	6 (9.1)	0.034
EBL (ml)	467 ± 47	392 ± 38	0.22
Intravenous crystalloid (24 h)	3,022 ± 422	3,044 ± 365	0.749
Total phenylephrine dose (mg)	1.4 (1.2, 1.8)	0 (0, 0)	<0.0001
Rescued metaraminol (mg)	0 (0, 0.5)	1 (0.5, 1.5)	<0.0001
pH value of umbilical arterial blood	7.30 ± 0.04	7.30 ± 0.03	0.339

Data are presented as n (%), mean ± SD or median (IQR).

**FIGURE 2:**
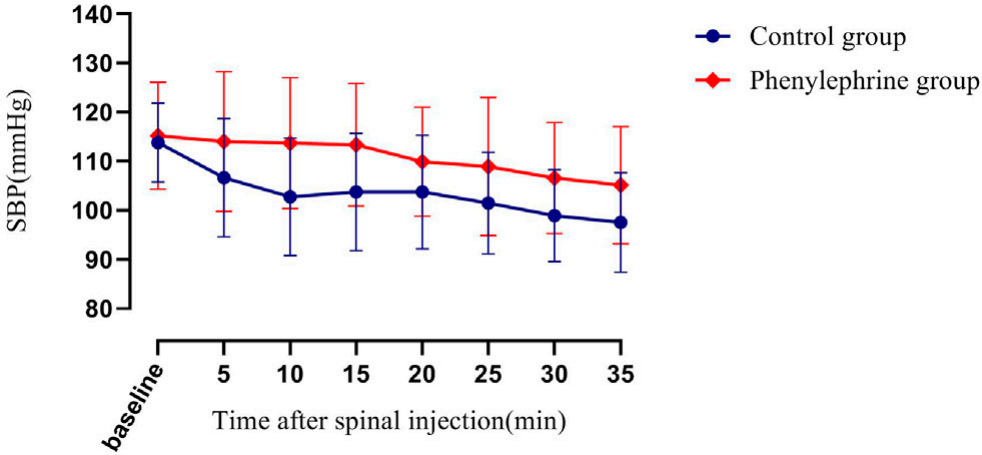
Baseline systolic blood pressure (SBP) and the SBP in the first 35 min after spinal induction is presented for the two groups. The area under the curve (mean ± SD) differed significantly between the two groups (4,930 ± 131 and 4,608 ± 113 min × mm Hg in the phenylephrine and control groups, respectively, *p =* 0.0033).

**FIGURE 3:**
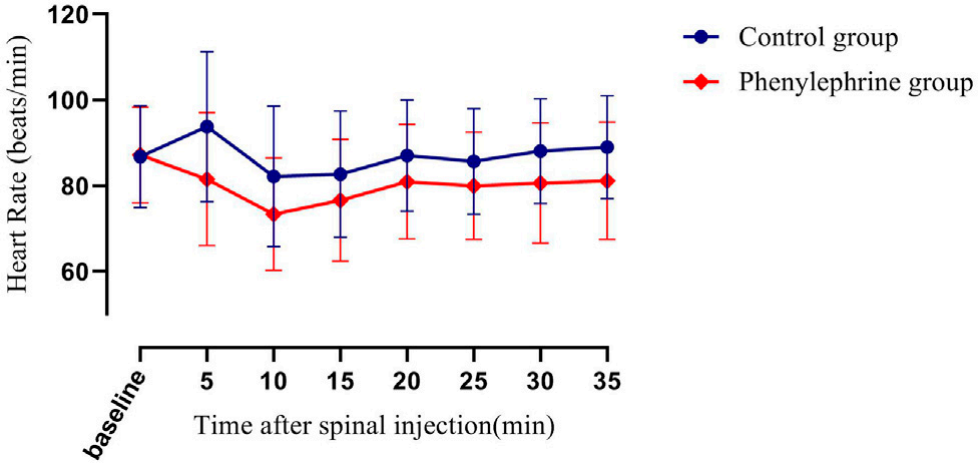
The baseline heart rate and the heart rate in the first 35 min after spinal induction is presented for the two groups. The area under the curve (mean ± SD) was significantly different when compared between the two groups (3,599 ± 141 and 3,927 ± 144 min × beats/min in the phenylephrine and control groups, respectively, *p =* 0.0003).

The baseline SBP and the SBP in the first 35 min after the induction of spinal anesthesia in the two groups are shown in Figure 2. The area under the curve (mean ± SD) differed significantly between the two groups (4,930 ± 131 and 4,608 ± 113 min × mmHg in the phenylephrine and control groups, respectively, *p =* 0.0033). Furthermore, the baseline heart rate, and the heart rate in the first 35 min after spinal induction, is presented in Figure 3. The area under the curve (mean ± SD) was significantly different when compared between the two groups (3,599 ± 141 and 3,927 ± 144 min × beats/min in the phenylephrine and control groups, respectively, *p =* 0.0003). SBP at the time of uterine incision was significantly higher in the phenylephrine group than in the control group (113.8 ± 13.5 mmHg *vs.* 102 ± 10.6 mm Hg, *p* < 0.0001).

Intraoperative adverse effects were shown by treatment group in Table 3. The incidence of hypotension was significantly higher before neonatal delivery in the control group (77.3%) than in the phenylephrine group (16.7%; *p* < 0.0001). Three patients experienced hypertension in the phenylephrine group and none in control group, with no significant difference between the groups. The incidence of bradycardia, nausea, flushing, and chest pain were also similar. No patients experienced vomiting or dyspnea in this study. Finally, the pH value of umbilical arterial blood was similar between the two groups.

**TABLE 3:** Comparison of side effects and SBP at time of uterine incision between two groups during CD.

	Phenylephrine group	Saline group	*p* Value
Hypotension			
Period from intrathecal injection to delivery	11 (16.7)	51 (77.3)	<0.0001
Period from intrathecal injection to surgery completed	41 (62.1)	60 (90.1)	0.0002
Hypertension	3 (4.5)	0 (0)	0.2443
Bradycardia	8 (12.1)	5 (7.6)	0.5607
Nausea	6 (9.1)	8 (12.1)	0.7785
Flushing	8 (12.1)	6 (9.1)	0.7785
Shivering	6 (9.1)	9 (13.6)	0.5847
SBP at time of uterine incision	113.8 ± 13.5	102 ± 10.6	<0.0001

Data are presented as n (%), mean ± SD or median (IQR).

## Discussion

This study demonstrated that a higher total oxytocin dose was required for women receiving a prophylactic phenylephrine infusion to prevent post-spinal hypotension during elective CD, than in a saline placebo-controlled infusion group. More patients in the phenylephrine group required secondary uterotonic agents; however, there was no difference in EBL between the intervention and control groups. To our knowledge, this is the first study that explores the effect of prophylactic phenylephrine on the dose requirement of oxytocin during CD.

The presence of adrenoceptors *β*_2_ receptors distributed in the cell membrane of the uterine smooth muscle has been well described. When this pathway is activated, intracellular cAMP levels will increase, thus decreasing the intracellular calcium level, causing uterine smooth muscle to relax, and inhibiting uterine contraction (Bóta et al., 2015). Phenylephrine is generally considered to be a pure *α*_1_ receptor agonist, which rarely acts on *β*-receptors and therefore plays no role on uterine relaxation during CD. Nevertheless, recent research reported that phenylephrine (as an ingredient of cold medication) can suppress uterine contraction in mice uteri through *β*_2_-receptor activity (Chen et al., 2018). In response to this, we designed this study, which demonstrated that the continuous infusion of phenylephrine to prevent post-spinal hypotension can increase the need of oxytocin to treat uterine atony. Our results provide evidence that phenylephrine prophylaxis may adversely affect uterine contractions. This has immediate practical implications; obstetricians and obstetric anesthesiologists must pay close attention to uterine tone for patients receiving a phenylephrine infusion for the prevention of post-spinal hypotension and increase the dosing of oxytocin where needed.

In this study, we adopted a continuous prophylactic phenylephrine infusion to prevent post-spinal hypotension. The use of this management strategy is now well documented and has the advantages of decreasing post-spinal hypotension and subsequent nausea and vomiting (Allen et al., 2010; Bishop et al., 2017; NganKee, 2017). A continuous infusion of phenylephrine to stabilize hemodynamics may increase the total delivery of phenylephrine during CD, and inevitably increase the plasma concentration of the drug. We hypothesize a relatively high plasma concentration of phenylephrine created by the continuous infusion activates *β*_2_ adrenoceptors, both inhibiting uterine contractions and increasing the need for exogenous oxytocin administration. However, in practice, many anesthesiologists may choose a single dose of phenylephrine to treat or prevent post-spinal hypotension, thus generating a lower plasma concentration. Further clinical studies are now needed to determine whether a single dose will have the same effect as continuous infusion of phenylephrine.

In this study, the average EBL in the 24-h after surgery in the phenylephrine group was 75 ml more than that in the control group. Although this was not significant, this study did not have sufficient power to detect a difference in EBL and a Type II error may have occurred. However, the SBP at the time of uterine incision in the phenylephrine group was higher than in the control group, thus suggesting that phenylephrine increases the perfusion pressure to the uterus by maintaining cardiac preload. This may be a factor underlying the increased amount of bleeding in the phenylephrine group. Therefore, subsequent clinical interventions, such as additional oxytocin, are needed to deal with the cause of bleeding. Further research is now required to determine whether phenylephrine prophylaxis increases the risk of postpartum hemorrhage. The incidence of hypotension in phenylephrine group was increased (from 16.7 to 62.1%) after neonatal delivery. This may suggest that higher doses of oxytocin result in relatively instable hemodynamics.

Although our data have important implications for obstetric anesthesiologists, there are some limitations. First, there was no objective assessment criterion used for UT. Although a subjective assessment of UT was made, both the surgery and assessment of UT were performed by the same obstetrician, based on over 15 years of obstetric experience. This is likely to improve a reliable (and blinded) estimate of uterine tone and is in line with routine practice. Second, UT can be affected by various factors, many of which may remain unknown. Despite randomization, these factors may have introduced confounding to the results of this relative small, randomized study. Larger, multi-center clinical trials are now warranted to corroborate our results across varied settings. Third, the estimation of EBL was achieved by a simple formula that is associated with several limitations. For example, this method is unlikely to be sensitive to small changes in the volume of blood loss and changes in haematocrit may manifest after the end of surgery. However, these limitations are unlikely to have affected the primary outcome of this study. Finally, Our exclusion of patients with a BMI of >35 kg/m^2^ may limit the generalizability of our findings to populations with a significant proportion of obese patients.

In conclusion, Prophylactic infusion of phenylephrine used to prevent post-spinal hypotension during CD was associated with a higher dose of oxytocin. This has important clinical implications, as the suboptimal use of oxytocin is associated with an increased risk of postpartum hemorrhage and increased maternal morbidity and mortality. Further studies are needed to confirm these finding.

## Data Availability

The datasets presented in this study can be found in online repositories. The names of the repository/repositories and accession number(s) can be found in the article/Supplementary Material.
